# Comments on "Effects of Patient and Tumor Characteristics on Central Lymph Node Metastasis in Papillary Thyroid Cancer: a Guide for Selective Node Dissection"

**DOI:** 10.34172/aim.27317

**Published:** 2024-08-01

**Authors:** Hadi Raeisi Shahraki

**Affiliations:** ^1^Department of Epidemiology and Biostatistics, Faculty of Health, Shahrekord University of Medical Sciences, Shahrekord, Iran

 Recently, Altiner et alpublished an article entitled above and showed that gender (OR = 1.22), tumor subtype (OR = 0.44) and lymphovascular invasion (OR = 2.59) significantly increase the odds of central lymph node metastasis (CLNM), while intra-thyroidal localization of the tumor is not significantly associated with CLNM based on the multiple logistic regression.^1^ However, these findings are doubtful due to the following reasons:

- The estimated crude odds ratios are not true for women (correct OR = 0.51), lymphovascular invasion (correct OR = 138.19) and extracapsular invasion (correct OR = 8.87).
- In Table 2, for not applicable ORs, 95% confidence intervals were reported!
- The negative values in the confidence interval of odds ratios are definitely wrong because the odds ratio is always positive.
- The adjusted OR for lymphovascular invasion is 2.59, but the 95% confidence intervals range from 4.47 to 7.9 which is impossible.
- In the multiple logistic regression, just one OR was reported for each of the quantitative variables with more than two categories. Considering a category as the baseline, four ORs must be reported for intra-thyroidal localization and two ORs for tumor subtype. So, the current ORs are not valid.
- The confidence interval of OR for gender ranges from 0.99 to 1.55 which contains one and cannot be significant; so, *P* value = 0.025 is not true and the correct *P *value is greater than 0.05, indicating gender is not statistically significant.

I hope this note shows some deleterious effects of inappropriate statistical analysis in medical research.
